# Displacement Forces in Different Proximal Landing Zones for Thoracic Endovascular Aortic Repair: Towards Patient-Specific Assessment

**DOI:** 10.3390/bioengineering13080868

**Published:** 2026-07-27

**Authors:** Michele Conti, Valentina Ceserani, Irene Baroni, Daniele Bianchi, Nicoletta Curcio, Alessandro Reali, Ferdinando Auricchio, Francesco Secchi, Daniela Mazzaccaro, Giovanni Nano, Paolo Righini

**Affiliations:** 1 Department of Civil Engineering and Architecture, University of Pavia, 27100 Pavia, Italy; 2 3D and Computer Simulation Laboratory, IRCCS Policlinico San Donato, 20097 San Donato Milanese, Italy; nicoletta.curcio@grupposandonato.it; 3 Clinical Research Service, IRCCS Policlinico San Donato, 20097 San Donato Milanese, Italy; 4 Unit of Nonlinear Physics and Mathematical Models, Università Campus Bio-Medico di Roma, 00128 Rome, Italy; d.bianchi@unicampus.it; 5 Unit of Cardiovascular Imaging, IRCCS MultiMedica, 20099 Sesto San Giovanni, Italy; 6 Department of Biomedical Sciences for Health, University of Milan, 20133 Milan, Italy; 7 Operative Unit of Vascular Surgery, IRCCS Policlinico San Donato, 20097 San Donato Milanese, Italy

**Keywords:** TEVAR, patient-specific modeling, computational fluid dynamics, hemodynamics, aortic biomechanics, magnetic resonance imaging

## Abstract

Thoracic endovascular aortic repair (TEVAR) is one of the main treatments, together with open aortic repair, for thoracic aortic aneurysm. This procedure is associated with low procedural morbidity and mortality and provides satisfactory mid-term results. However, identifying the optimal location for device deployment before the operation remains essential. In previous studies, computer simulations were used to examine the hemodynamic stress linked to different areas of the arch, pinpointing regions unsuitable for TEVAR delivery. Computational fluid dynamics (CFD) simulations suggest that zone 3 poses the most challenges for delivering endograft in type III arches, as it is considered a hostile region from biomechanical and hemodynamic perspectives. Morphologically, type III aortic arch shows a more pronounced curvature and a lower position of the arch, resulting in increased separation between the ascending and descending aorta. However, these studies utilized 3D geometrical models reconstructed from computed tomography angiography scans. Still, the flow boundary conditions were based on existing data rather than the specific hemodynamic characteristics of the patient. To bridge this gap, the present study uses MRI flow wave data to compute displacement forces (DFs) for each proximal landing zone. We found that zones 0 and 3 exhibited a high magnitude of displacement forces. Still, only zone 3 was identified as the most hemodynamically stressed area when the force was normalized for the lumen area. Furthermore, there was a significant discontinuity in the upward components of DFs between zone 2 and zone 3. The patient-specific assessment of displacement forces enabled us to identify suboptimal areas for TEVAR delivery, suggesting that personalized computer simulations could greatly enhance preoperative planning for TEVAR procedures.

## 1. Introduction

Thoracic Endovascular Aortic Repair (TEVAR) is now the preferred treatment for aortic aneurysms, even for low-risk patients. This is because it has a lower 30-day mortality rate and good mid-term results at 5 years in terms of survival compared to open surgery [1].

However, successful TEVAR treatment in the long term requires preoperative surgical planning [2]. The primary objective of preoperative TEVAR planning is to identify a suitable proximal landing zone (PLZ) to ensure a safe, feasible procedure with long-lasting results [3]. This requires a healthy aortic wall and a proper diameter and length (i.e., less than 40 mm and more than 20 mm, respectively) for successful endograft deployment [4]. 

To plan for endovascular surgery, it is crucial to take into account not only the geometric factors such as the location, angulation, and tortuosity of the aneurysm (especially in the case of aortic arch involvement) but also the impact of blood flow on the vessel wall and supra-aortic trunks [5].

Recent studies have used computer simulations to evaluate the hemodynamics of healthy and pathological aortic arches. The main objective of the CFD analysis is to identify the patient-specific biomechanical characteristics of the aortic arch and optimize the endoprosthesis placement in TEVAR procedures before the surgery to improve post-operative clinical outcomes. These simulations help assess the impact of hemodynamic forces on the proximal landing zone (PLZ) of the aortic arch. Specifically, previous studies using computational fluid dynamics (CFD) simulations have shown that zone 3 is the most challenging area for endograft deployment. This finding was obtained using 3D models reconstructed from computed tomography (CT) images of healthy patients, which were then applied to pathological patients. 

In particular, Marrocco-Trischitta and colleagues [6] proposed the Modified Arch Landing Areas Nomenclature (MALAN), which merges Ishimaru’s map with the Aortic Arch Classification [6]. In 2018 [7], a study utilized computational fluid dynamics (CFD) modelling to investigate how well the MALAN classification system could describe the strength and direction of displacement forces in proximal landing zones for TEVAR. The researchers examined CT angiography scans of healthy aortas with different arch shapes (Types I to III), five per group. They assessed the pulsatile displacement forces in specific areas of the aorta. They observed that in Types I and II arches, the equivalent surface traction (EST) did not change across proximal landing zones, whereas in Type III arches, EST increased towards more distal landing zones. Additionally, comparing EST between adjacent zones revealed that EST was greater in Type III than in Type II. The study suggested that these findings could have implications for clinical treatments using TEVAR but emphasized the need for further research to validate these results in post-operative patient analysis [7]. 

These findings were confirmed by identifying unfavorable landing zones for endograft deployment in diseased aortas. The study reviewed preoperative CT angiography scans of 10 patients scheduled for thoracic endovascular aortic repair. Findings showed severe angulation in certain landing areas and increased magnitude and unfavorable orientation of equivalent surface traction. Three of the ten cases experienced adverse events related to proximal endograft performance. The study confirmed that MALAN can effectively identify landing zones in hostile geometric and hemodynamic environments, thereby increasing the risk of poor endograft performance in diseased aortas [8]. Finally, this approach was applied to the bovine arch, demonstrating that this particular aortic arch anatomy exhibits a consistent fluid dynamic pattern. This pattern indicates an unfavorable biomechanical environment for endograft deployment in zone 3 [9]. 

The Modified Arch Landing Areas Nomenclature (MALAN) value for thoracic endovascular aortic repair (TEVAR) was assessed through a retrospective analysis of 359 patients. The study found that hostile landing areas (2/III and 3/III) were associated with poorer postoperative outcomes, whereas favorable landing areas (0/I–III, 1/I–III, 2/I–II, 3/I–II) were associated with better endograft performance. This suggests that the MALAN classification is a valuable tool for preoperative decision-making [10].

Prior studies in this area have used only patient-specific anatomical information from 3D models of the aorta generated from CT scans. Therefore, this study aims to validate those findings by conducting a patient-specific computational fluid dynamics (CFD) simulation. This simulation will utilize the geometric and hemodynamic data obtained from CT scans and phase-contrast magnetic (PC) resonance imaging (MRI).

## 2. Material and Methods

### 2.1. Study Cohort

The present study includes patients enrolled in the MALAN study (study number RF-2016-02363679) who met specific criteria. To be included, patients had to be 18 years or older, have a chronic thoracic aortic disease, and be eligible for surgical treatment with TEVAR. However, patients with a history of previous aortic arch endovascular surgical procedures, general contraindications to MRI or angiographic studies, suspected or confirmed pregnancy, or systemic conditions that were not compatible with the procedures were excluded from the study. These steps entail considerable computational time and cost, and not all clinical centers have access to PC-MRI of sufficient quality to obtain accurate flow waveforms. Moreover, the high prevalence of bovine arches mirrors the characteristics of the patient population enrolled during the study period rather than a selection bias.

### 2.2. Imaging and Data Processing

**Figure 1:**
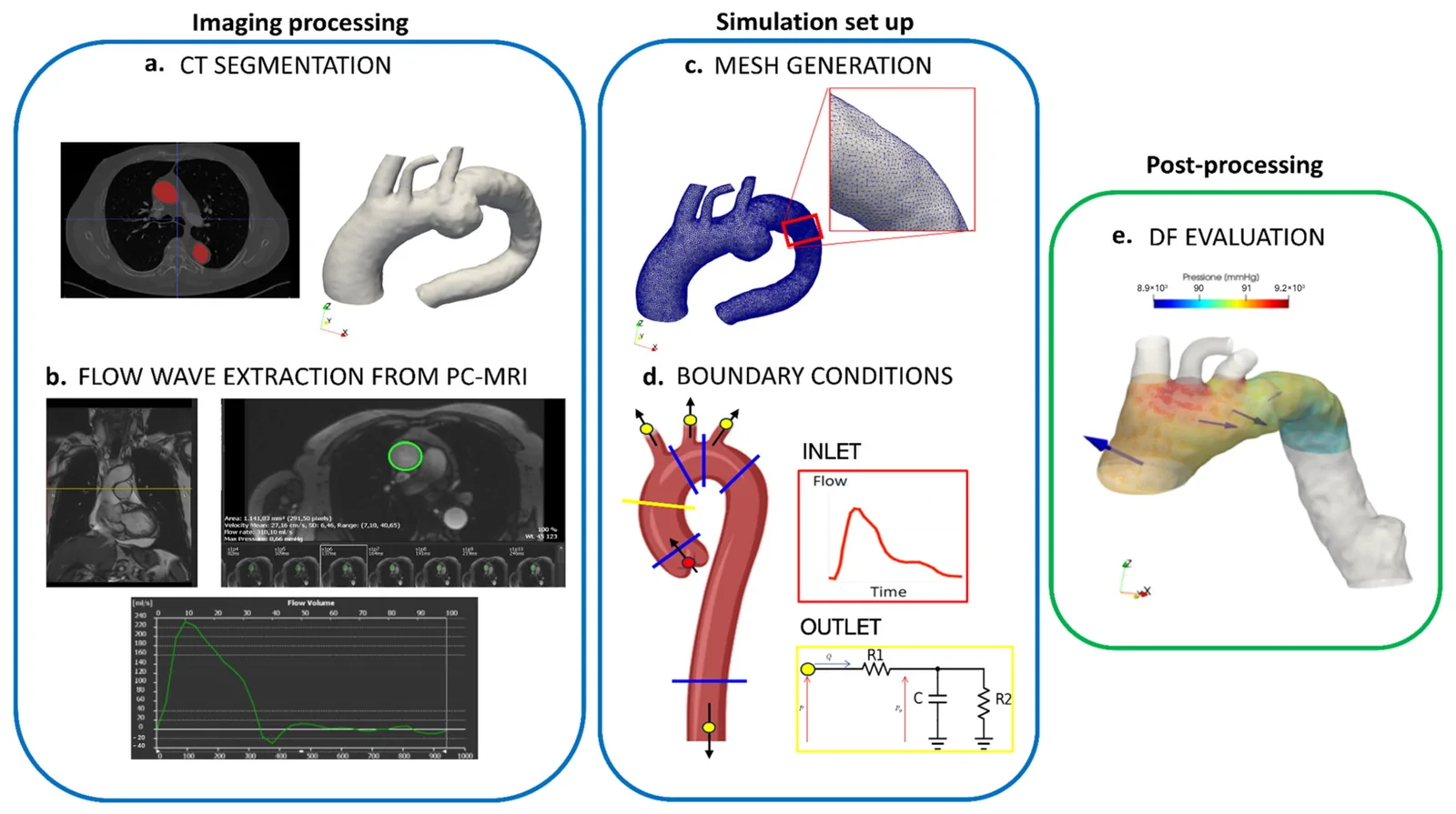
Workflow of patient-specific CFD simulation. Patient-specific imaging data are processed: CT was segmented to obtain the 3D reconstruction of the fluid domain (**a**), and PC-MRI sequences were analyzed to extract the flow wave of the aorta at different planes along the vessel’s centerline (**b**). The imaging processing allows the generation of the domain numerical mesh (**c**) and defines the model’s boundary conditions (**d**). Post-processing the patient-specific CFD simulation results provides for evaluating the displacement forces acting on each aortic arch zone (**e**).

All patients underwent CT and MRI scans. A 16-slice unit with 150 mAs and 110 kVp was used for CT. The acquisition thickness was 5 mm with a pitch of 1.5 and a reconstruction thickness of 1.2 mm. CT images were segmented before and after intravenous administration of 100 mL of iodinated contrast material to define a 3D patient-specific reconstruction of the aortic lumen that represented the fluid domain for CFD simulation, illustrated in Figure 1a. A 1.5-T unit with 40-mT/m gradient power (Magnetom, Siemens, Erlangen, Germany) and a four-channel cardio-thoracic coil were used for MRI. ECG-triggered, free-breathing through the plane, and in-plane PC-MRI sequences were performed for phase-velocity mapping of the aortic and the flow of the branches. The following technical parameters were set: repetition time (TR, i.e., the time between successive radiofrequency pulses)/echo time (TE, i.e., the time between the radiofrequency pulse and the signal acquisition) = 4/3.2 ms, thickness 5 mm, velocity encoding from 150 to 350 ms, and temporal resolution 41 ms. Six axial plane slices along the aortic arch were acquired. The Medis software (https://medisimaging.com/) was used to post-process PC-MRI sequences and accurately quantify each plane’s patient-specific blood flow wave, as shown in Figure 1b. These flow waves, along with the patient’s systolic and diastolic pressure values, were used to calibrate the parameters of the Windkessel model based on the methodology described in the study by Romarowski et al. [11].

### 2.3. Computational Simulation

The time evolution of blood flow in the aortic arch is modelled through the Navier–Stokes equations, assuming blood as an incompressible Newtonian fluid with a density of 1.06 g/cm^3^ and viscosity of 0.04 Poise. After defining the fluid domain discretization (Figure 1c), the Navier–Stokes equations were solved using the finite element method through the SimVascular software simulation package (v2019.08.0915) and considering six heartbeats. Cardiac output specific to the patient is determined using a patient-specific flow wave extracted from the PC-MRI sequence corresponding to a transverse plane of the proximal ascending aorta. A Dirichlet boundary condition with a parabolic velocity profile is imposed, as shown in Figure 1d. The three-element Windkessel circuit models all aortic outlets, calibrated using patient-specific flow waves extracted from PC-MRI and patient-specific arterial pressure, as illustrated in Figure 1d. The flow waves extracted from the sections immediately before and after each supra-aortic vessel are subtracted to derive the flow wave that characterises each outlet. The optimal values of the Windkessel circuit are computed through an iterative process of error minimization [12]. In some cases, the quality of the PC-MRI data did not allow the extraction of reliable patient-specific flow boundary conditions; in these instances, boundary conditions were approximated using data from matched patients with similar age, sex, and aneurysm location. Simulations were conducted, and the results were analyzed to determine the displacement force (DF) values at all proximal landing zones (PLZs) previously identified in Figure 1e. The DFs were computed by integrating the aortic surface pressure at the systolic peak along the aortic wall at each PLZ. This study did not consider wall shear stress; only geometric differences were considered. Hence, the DF values were normalized concerning the surface area of each zone to obtain the equivalent surface traction (EST) parameter. The study evaluated the magnitude and direction of DF and EST for all patients enrolled. The results were compared with different PLZ to identify any adverse zones for TEVAR delivery.

### 2.4. Statistical Analysis

We compared the magnitude and components of DF between PLZ. The comparison was done using the Wilcoxon Rank test for paired data. Additionally, we compared the EST in the same way. All results are presented as median and interquartile range. Statistical significance was assumed at *p*-value < 0.05.

## 3. Results

The study examined the first four landing zones of the aortic arch (excluding Zone 1 in patients with a bovine arch).

The study included 13 patients (mean age 73.6 ± 7.6 years) enrolled between 2019 and 2021. The limited sample size reflects the highly experimental and personalized nature of the analysis: each case required a full three-dimensional reconstruction of the aorta, patient-specific flow acquisition through PC-MRI, and an individualized CFD simulation followed by extensive post-processing. These steps demand considerable computational time and resources, which justifies the restricted number of patients in this preliminary investigation.

The magnitudes and directions of the DFs were calculated for each zone, including the upward and sideways components. To understand the differences in the geometrical attributes of each section, the DFs were normalized concerning the area of each zone. Post-processing the simulation results allowed for the computation of the magnitude and direction of the DFs on each PLZ.

**Figure 2:**
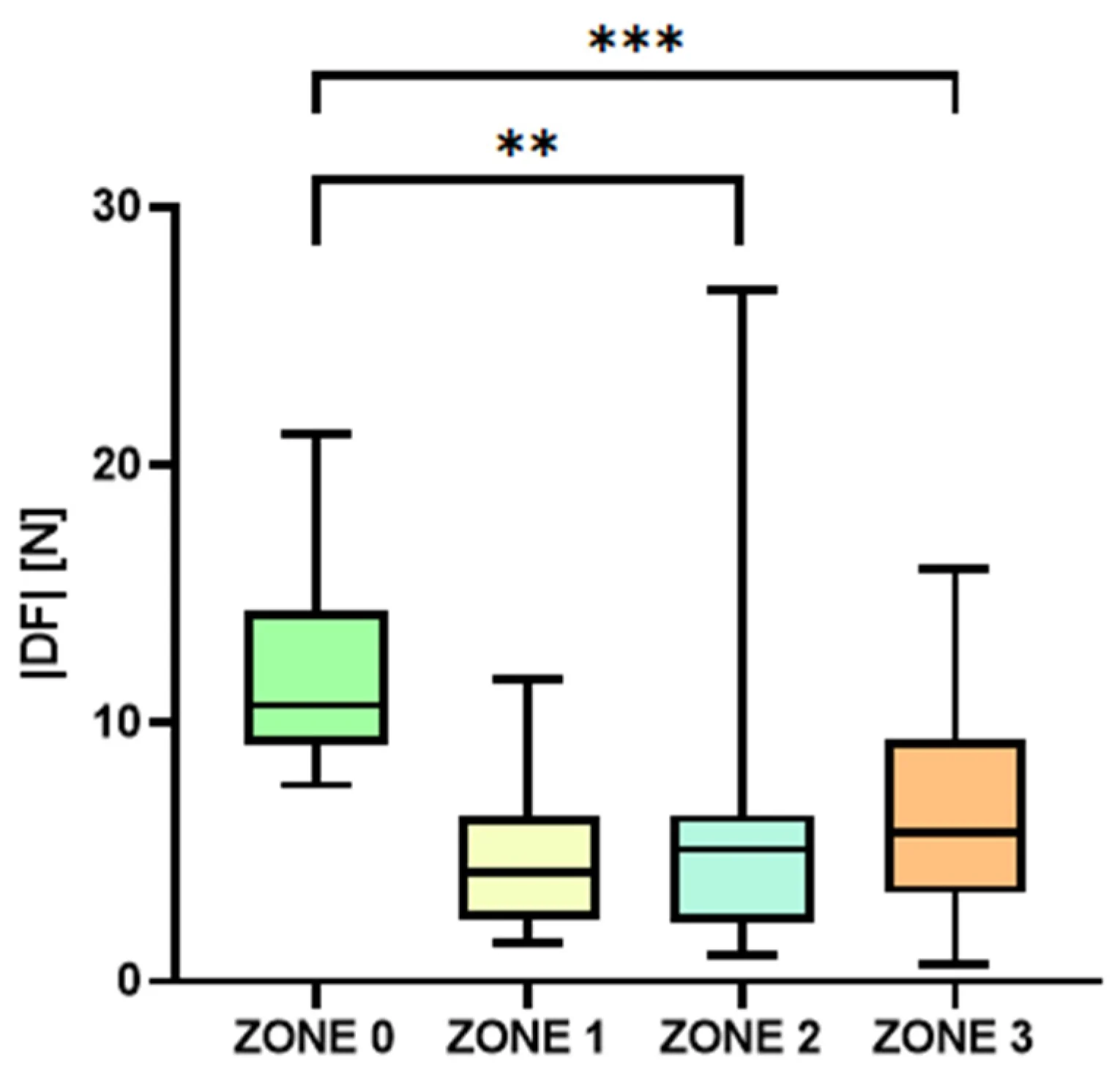
Boxplots of DFs magnitude for each zone are shown. Only statistically significant differences are highlighted (**, ***).

The results are presented in Table 1, and the boxplots of the DFs are shown in Figure 2. The highest magnitude value of DFs was found in Zone 0 (10.7 N; IQR: 9.2–14.4 N), which was found to be significantly different from Zone 2 (5.1 N; IQR: 2.3–6.5 N; *p* = 0.0061) and Zone 3 (5.7 N; IQR: 3.4–9.4 N; *p* < 0.001). No significant differences were found between Zone 2 and Zone 3.

**Figure 3:**
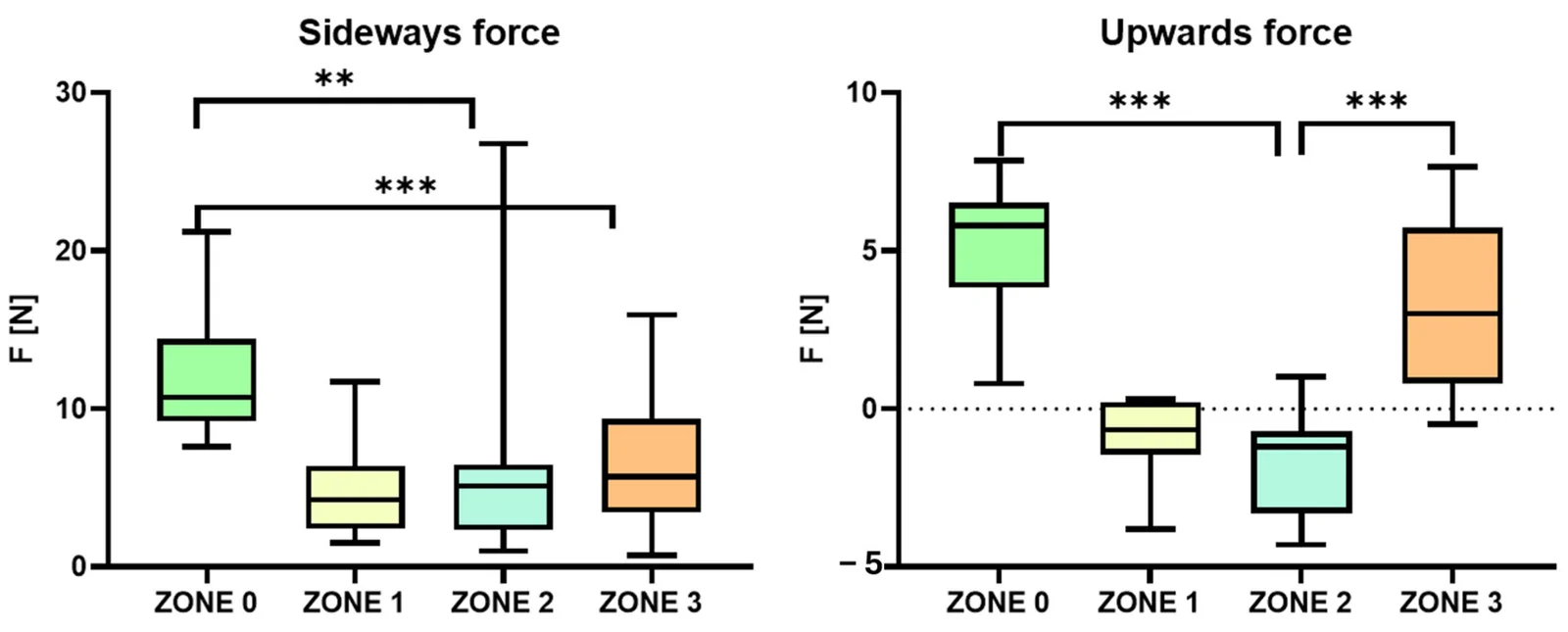
Box plot of the lateral component of the DF vector on the left and the vertical component on the right, relating to the 4 areas of the aortic arch considered. The groups between which there is a statistically significant difference are highlighted. (**, ***).

DF components were also calculated for each vector according to the two main directions (‘sideways’ and ‘upward’) and then compared between landing zones. Sideways forces were higher than upward forces, as shown in Figure 3. The data show a strong statistically significant difference between Zone 2 (−1.2 N; IQR: −3.3–0.7 N) and Zone 3 (3 N; IQR: 0.8–5.7 N) for upward components, indicating a strong discontinuity in the direction of DFs between these two zones. 

The sideways forces are reported in Table 2, while the upward forces are reported in Table 3.

**Figure 4:**
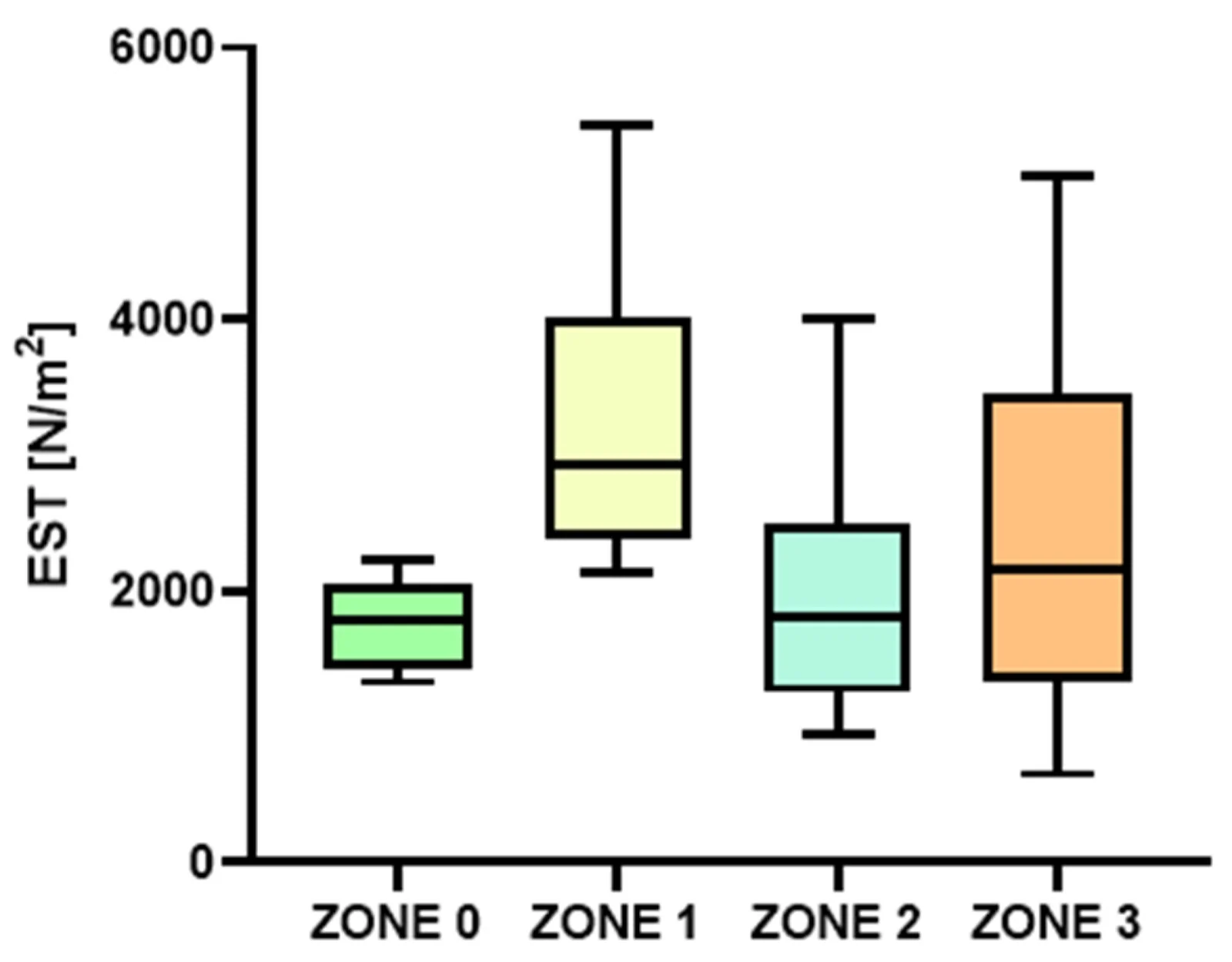
Shows Boxplots of Equivalent Surface Traction (EST) for each zone.

The magnitude value of DFs was normalized to the area of each zone to account for the impact of geometric differences. Table 4 shows the evaluated EST values. As shown in Figure 4, zone 1 has the most higher EST value; this result is only related to a subgroup of the population (*n* = 8) with a non-bovine arch. 

In general, the population shows more significant values of EST at more distal zones, indicating that zones 2 and 3 are subjected to relevant hemodynamic conditions. Zone 3, in particular, has a more considerable EST value (2155 N/m^2^; IQR:1330–3462 N/m^2^) than zone 2 (1818 N/m^2^; IQR: 1253–2503 N/m^2^) but without a statistically significant difference (*p* > 0.05).

To better understand the clinical significance of Computational Fluid Dynamics (CFD) analysis in preoperative Thoracic Endovascular Aortic Repair (TEVAR) planning, a case-based comparison was conducted. This comparison analyzed the difference between planning based solely on CT imaging (anatomical planning) and planning informed by patient-specific CFD assessment (CFD-informed assessment) (Table 5). Specifically, for patients with available follow-up data, the intraoperative landing zone (LZ) chosen using CT-based planning was compared against the optimal LZ identified by CFD analysis. The optimal LZ was defined as the zone exhibiting the lowest magnitude of displacement force (DF).

In 5 out of 9 patients (Patients 02, 03, 06, 07, and 09), the CFD-derived optimal landing zone differed from the one selected based on anatomical criteria alone. In these cases, CFD suggested either a more distal (e.g., Patient 02: LZ2 → LZ3) or more proximal (e.g., Patients 07 and 09: LZ0 → LZ1) sealing zone. Notably, all five patients presented adverse findings at follow-up, including bird-beak configuration and/or Type IA endoleak, which are issues typically associated with suboptimal landing zone selection.

Conversely, in all patients in whom CFD-informed and CT-based planning were concordant (e.g., Patients 01, 05, 10, and 13), no complications were observed at follow-up.

## 4. Discussion

The endovascular repair of aortic arch disease is a complex procedure, and ongoing research is being conducted to understand its long-term outcomes. One key area of improvement is enhancing the preoperative decision-making process through better preoperative endovascular planning [13]. Despite numerous studies, preoperative planning for TEVAR still relies solely on static imaging protocols. Implementing patient-specific hemodynamic modelling into TEVAR planning presents challenges. 

In the present study, thirteen patients with aortic thoracic aneurysms underwent patient-specific CFD simulations to assess the magnitude and direction of DFs on different areas of the aortic arch. Both the geometrical model and flow boundary conditions were patient-specific. The results showed that zone 0 exhibited the highest DF magnitude, suggesting that hemodynamic loads may also be relevant for TEVAR involving the ascending aorta and proximal arch. Nevertheless, zone 3 emerged as the most biomechanically hostile landing zone because of its unfavorable force orientation and greater geometric complexity, despite being the most common proximal anchoring site for current stent-graft designs. 

Moreover, integrating patient-specific CFD simulations into preoperative planning could improve the selection of both the device and the precise deployment point, even within the same zone 3, thus making TEVAR planning more predictive and personalized. The results of this study also suggest that more proximal landing zones (such as zones 0 or 1), although associated with greater surgical complexity and the need for more demanding techniques, exhibit higher, more uniform, and more predictable displacement forces. From a biomechanical perspective, this implies that, when technically feasible and supported by suitable device design, proximal anchoring may provide greater long-term stability of the endograft. 

It is important to gather additional evidence to conclude how patient-specific flow impacts the results of aortic hemodynamics displacement forces calculated by CFD. In addition, the present cohort focused on a specific class of thoracic aortic pathology treated with TEVAR and included patients with a relatively narrow age range (73.6 ± 7.5 years). This homogeneity does not allow for assessing the potential effect of age-related changes in aortic wall properties on the computed displacement forces. However, since both geometry and boundary conditions were patient-specific, the influence of age is indirectly incorporated in the simulations. Future studies with larger and more heterogeneous populations will be useful to explicitly evaluate age-dependent biomechanical differences. 

The study has also highlighted the limitations of obtaining medical images required to extract flow waves to define patient-specific boundary conditions. Performing phase-contrast MRI is challenging in several medical centers, which hinders the ability to conduct multi-center investigations and limits the number of eligible patients. This suggests the need for a more precise and defined protocol for acquiring clinical data and a thorough assessment of the impact of using patient-specific flow data in patient computational fluid dynamics analysis of the aorta [14].

Furthermore, these types of studies can be applied to other aortic regions. Belvroy et al. [15] extended this approach to the descending thoracic aorta (DTA) to investigate how increased twisting in DTA affects the distribution of hemodynamic forces on different segments in patients with DTA aneurysms. Patients were divided into low-, moderate-, and high-twisting groups. Computational fluid dynamics simulations showed that high twisting led to significantly higher forces in the distal segments of the DTA, potentially increasing the risk of complications during TEVAR [15]. Nevertheless, patient-specific computational tools can be used to analyze displacements within the heartbeat and changes in shape over time of endograft used for repairing abdominal aortic aneurysms. These tools can also be used to relate these changes to CFD, as proposed by Domanin et al. [16]. They investigated two cases treated with different endografts using dynamic CTA at 1, 12, and 60 months. The results showed minimal translations without deformation in one endograft, while the other showed no deformation. CFD analysis revealed changes in velocity patterns and flow disturbances consistent with the displacement analysis, providing valuable insights for physicians and manufacturers regarding endograft behavior over time [16], potentially leading to the design of next-generation stent-grafts, optimized for challenging landing zones.

Beyond the methodological contribution, the present findings also carry relevant implications for clinical practice. The integration of patient-specific CFD simulations into preoperative TEVAR planning has the potential to enhance clinical decision-making beyond conventional anatomical assessment. By quantifying local hemodynamic loads and DFs, this approach enables a more objective identification of biomechanically hostile landing zones, particularly in complex arch morphologies such as Type III and bovine arches. The results obtained in this study suggest that CFD-informed analysis can complement CT-based planning by highlighting regions at increased risk of endograft migration or malapposition. Ultimately, incorporating such computational tools into routine planning could foster a more personalized and predictive strategy for TEVAR, improving both procedural safety and long-term outcomes.

Importantly, the case-based comparison reported in Table 5 provides a first practical demonstration of how patient-specific CFD analysis may complement conventional TEVAR planning. In a subset of patients, the CFD-derived optimal landing zones differed from those selected based on CT-based anatomical assessment alone, suggesting that geometric criteria alone may not fully capture the underlying biomechanical environment. Although postoperative outcomes are also influenced by device characteristics, sizing strategy, and deployment technique, the observed association between discordant planning and adverse findings at follow-up (e.g., bird-beak configuration and Type IA endoleak) supports the hypothesis that patient-specific hemodynamic information may provide additional value for identifying biomechanically unfavorable landing zones. Conversely, concordance between CFD-informed and anatomical planning was associated with uneventful follow-up. While these findings should be interpreted with caution because of the limited sample size and heterogeneous endograft platforms, they suggest that CFD-informed analysis may complement current imaging-based planning by adding a quantitative biomechanical perspective to clinical decision-making. 

However, it is worth noting that one of the main issues is the high computational cost and the misalignment with the clinical timeline. Retrieving data from medical images, setting up the simulation, and post-processing take much time. It is not automated enough to provide real-time support during the planning process. This means that these approaches apply only to elective surgery [17]. However, efforts are being made to address these limitations. For instance, there are attempts to use neural networks to automatically segment the aorta in CTA scans [18] and to employ methods that can act as substitutes for CFD computations, as proposed by Sturla and colleagues [19], which show promise.

Although our findings show that displacement forces in the ascending aorta and proximal arch are higher in magnitude, they are also more uniform and predictable; in perspective, this biomechanical profile may support the development of safer proximal sealing strategies, informed by patient-specific simulations and tailored device design—including future applications in ascending aorta endografting [20]. Such insights could help balance procedural feasibility and long-term stability in next-generation TEVAR approaches.

## 5. Conclusions

The evaluation of displacement forces about the geometric characteristics of PLZs has helped identify specific suboptimal areas for endoprosthesis delivery. The patient-specific, in silico simulations developed in this study could improve preoperative decision-making by optimizing the preoperative endovascular planning. This could help clinicians refine landing-zone selection and complement the choice of endograft with suitable mechanical characteristics for the patient’s biomechanical environment. Applying this simulation to a sample of patients during planning for TEVAR treatments may help reduce the risk of complications, particularly endoprosthesis migration, in the general population.

## Figures and Tables

**Table 1:** The magnitude of the DFs [N] relating to each landing zone for each patient considered is reported. Zone 1 cannot be identified for patients with bovine arch.

DF Magnitude [N]	Zone			
Patient ID	0	1	2	3
01	9.3	bovine	1.4	2.3
02	10.1	bovine	5.4	2.7
03	7.6	1.5	1	0.7
04	9.1	2.9	6.3	8.6
05	13.5	6.6	5.2	6
06	10.9	bovine	2.2	4.3
07	21.2	5.7	26.8	10.9
08	10.7	bovine	2.4	5.4
09	15.96	11.71	18.79	15.95
10	10.57	2.21	5.12	10.13
11	8.28	bovine	3.43	7.12
12	13.44	5.43	2.75	4.09
13	15.35	3.01	6.62	5.72

**Table 2:** The values of the lateral (sideways) component of the DF vector relating to each landing zone for each patient considered are reported. Zone 1 cannot be identified for patients with bovine arch.

Sideway Component [N]	Zone			
Patient ID	0	1	2	3
01	6.7	bovine	0.9	2.3
02	6.7	bovine	3.3	2.4
03	3.7	1.46	0.8	0.6
04	7.5	2.82	4.6	7.1
05	13.2	6.59	5.0	5.9
06	9.2	bovine	2.12	2.5
07	20.5	5.71	26.8	10.9
08	8.5	bovine	2.1	4.6
09	15.2	11.07	18.8	14.0
10	8.4	2.11	3.5	6.9
11	5.6	bovine	3.4	2.8
12	10.9	5.16	0.5	2.0
13	15.1	3.01	5.9	4.9

**Table 3:** The values of the vertical (upward) component of the DF vector relating to each landing zone for each patient considered are reported. Zone 1 cannot be identified for patients with bovine arch.

Upward Component [N]	Zone			
Patient ID	0	1	2	3
01	7.3	bovine	1	0.5
02	0.8	bovine	−4.3	1.2
03	6.6	0.3	−0.6	0.4
04	5.2	−0.7	−4.3	4.9
05	2.8	−0.7	−1.5	1.1
06	5.8	bovine	−0.7	3.5
07	5.4	0.3	−0.9	−0.5
08	6.4	bovine	−1.2	2.9
09	4.7	−3.811	−0.9	7.6
10	6.4	−0.64	−3.54	7.4
11	6.1	bovine	−0.72	6.5
12	7.8	−1.69	−2.7	3.6
13	2.9	−0.03	−3.07	3.0

**Table 4:** The values of EST relating to each landing zone for each patient considered are reported. Zone 1 cannot be identified for patients with bovine arch.

Equivalent Surface Traction [N/m^2^]	Zone			
Patient ID	0	1	2	3
01	1332.8	bovine	937.5	1016.4
02	1390.2	bovine	2417.1	1103.7
03	2153.3	2902.7	1109.9	653.6
04	2232.3	2238.5	2589.2	4411.2
05	1913.7	3789.5	1383.6	2131.8
06	1478.9	bovine	1582.1	2930.2
07	2200.4	4087.7	3997.7	2155.2
08	1782.2	bovine	1690.5	1555.3
09	1765.44	5427.18	3650.4	5065.92
10	1961.98	2141.97	1818.11	2312.89
11	1334.41	bovine	2336.67	3992.91
12	1669.62	2962.55	1121.85	1787.19
13	1860.82	2780.45	1869.39	2789.01

**Table 5:** Case-based comparison between CT-based anatomical planning and CFD-informed planning for TEVAR.

Patient ID	Implanted Stent-Graft	Intraoperative LZ (CT-Based)	Best LZ According to CFD-Informed Planning	Follow-Up 6 Months (CT-Based)
01	Metronic Valiant Navion	LZ 2	LZ 2	No complication
02	MedTronic CoveredSeal	LZ2	LZ3	Persistent flow in the left subclavian artery with a bird-beak
03	17/0	LZ0 with debranching SAT	LZ3	Bird-beak
05	Gore C-TAG (34320E4)	LZ2	LZ2	No complication
06	Cook ZTA	LZ0 with chimney on SAT	LZ2	Endoleak Type IA and bird-beak
07	Gore C-TAG	LZ0 with chimney on SAT	LZ1	Endoleak Type IA and bird-beak
09	Cook ZTA	LZ0 with chimney on SAT	LZ1	Bird-beak
10	Gore C-TAG	LZ2	LZ2	No complication
13	Cook ZTA	LZ2	LZ2	No complication

## Data Availability

The data presented in this study are available on reasonable request from the corresponding author. The data are not publicly available because they include clinical imaging data that could compromise patient confidentiality and are subject to institutional data-sharing restrictions.

## References

[1] Böckler D., Brunkwall J., Taylor P.R., Mangialardi N., Hüsing J., Larzon T., Hyhlik-Dürr A., Gawenda M., Clough R., Ronchey S. (2016). Thoracic Endovascular Aortic Repair of Aortic Arch Pathologies with the Conformable Thoracic Aortic Graft: Early and 2-Year Results from a European Multicentre Registry. Eur. J. Vasc. Endovasc. Surg..

[2] Erbel R., Aboyans V., Boileau C., Bossone E., Di Bartolomeo R., Eggebrecht H., Evangelista A., Falk V., Frank H., Gaemperli O. (2014). 2014 ESC Guidelines on the Diagnosis and Treatment of Aortic Diseases. Kardiol. Pol..

[3] Isselbacher E.M., Preventza O., Hamilton Black J., Augoustides J.G., Beck A.W., Bolen M.A., Braverman A.C., Bray B.E., Brown-Zimmerman M.M., Chen E.P. (2022). 2022 ACC/AHA Guideline for the Diagnosis and Management of Aortic Disease: A Report of the American Heart Association/American College of Cardiology Joint Committee on Clinical Practice Guidelines. Circulation.

[4] Ishimaru S. (2004). Endografting of the Aortic Arch. J. Endovasc. Ther..

[5] van Bogerijen G.H., Tolenaar J.L., Conti M., Auricchio F., Secchi F., Sardanelli F., Moll F.L., van Herwaarden J.A., Rampoldi V., Trimarchi S. (2013). Contemporary Role of Computational Analysis in Endovascular Treatment for Thoracic Aortic Disease. Aorta.

[6] Marrocco-Trischitta M.M., de Beaufort H.W., Secchi F., van Bakel T.M., Ranucci M., van Herwaarden J.A., Moll F.L., Trimarchi S. (2017). A Geometric Reappraisal of Proximal Landing Zones for Thoracic Endovascular Aortic Repair According to Aortic Arch Types. J. Vasc. Surg..

[7] Marrocco-Trischitta M.M., van Bakel T.M., Romarowski R.M., de Beaufort H.W., Conti M., van Herwaarden J.A., Moll F.L., Auricchio F., Trimarchi S. (2018). The Modified Arch Landing Areas Nomenclature (MALAN) Improves Prediction of Stent Graft Displacement Forces: Proof of Concept by Computational Fluid Dynamics Modelling. Eur. J. Vasc. Endovasc. Surg..

[8] Marrocco-Trischitta M.M., Romarowski R.M., de Beaufort H.W., Conti M., Vitale R., Secchi F., Auricchio F., Trimarchi S. (2019). The Modified Arch Landing Areas Nomenclature Identifies Hostile Zones for Endograft Deployment: A Confirmatory Biomechanical Study in Patients Treated by Thoracic Endovascular Aortic Repair. Eur. J. Cardio-Thorac. Surg..

[9] Marrocco-Trischitta M.M., Romarowski R.M., Alaidroos M., Sturla F., Glauber M., Nano G. (2020). Computational Fluid Dynamics Modeling of Proximal Landing Zones for Thoracic Endovascular Aortic Repair in the Bovine Arch Variant. Ann. Vasc. Surg..

[10] Marrocco-Trischitta M.M., de Beaufort H.W., Piffaretti G., Bonardelli S., Gargiulo M., Antonello M., Van Herwaarden J., Boveri S., Bellosta R., Trimarchi S. (2020). The Modified Arch Landing Areas Nomenclature Predicts Proximal Endograft Failure after Thoracic Endovascular Aortic Repair. Eur. J. Cardiothorac. Surg..

[11] Romarowski R.M., Lefieux A., Morganti S., Veneziani A., Auricchio F. (2018). Patient-Specific CFD Modelling in the Thoracic Aorta with PC-MRI–Based Boundary Conditions: A Least-Square Three-Element Windkessel Approach. Int. J. Numer. Methods Biomed. Eng..

[12] Domanin M., Bissacco D., Romarowski R.M., Conti M., Auricchio F., Ferraresi M., Trimarchi S. (2021). Drag Forces after Thoracic Endovascular Aortic Repair: General Review of the Literature. Ann. Vasc. Surg..

[13] Mandigers T.J., Ramella A., Bissacco D., Domanin M., van Herwaarden J.A., Heijmen R., Luraghi G., Migliavacca F., Trimarchi S. (2023). Thoracic Stent Graft Numerical Models to Virtually Simulate Thoracic Endovascular Aortic Repair: A Scoping Review. Eur. J. Vasc. Endovasc. Surg..

[14] Antonuccio M.N., Mariotti A., Fanni B.M., Capellini K., Capelli C., Sauvage E., Celi S. (2021). Effects of Uncertainty of Outlet Boundary Conditions in a Patient-Specific Case of Aortic Coarctation. Ann. Biomed. Eng..

[15] Belvroy V.M., Romarowski R.M., van Bakel T.M., van Herwaarden J.A., Bismuth J., Auricchio F., Moll F.L., Trimarchi S. (2020). Impact of Aortic Tortuosity on Displacement Forces in Descending Thoracic Aortic Aneurysms. Eur. J. Vasc. Endovasc. Surg..

[16] Domanin M., Piazzoli G., Trimarchi S., Vergara C. (2020). Image-Based Displacements Analysis and Computational Blood Dynamics after Endovascular Aneurysm Repair. Ann. Vasc. Surg..

[17] Song J., Gao S., Xie E., Wang W., Dai L., Zhao R., Zhou C., Qiu J., Yu C. (2023). Systematic Review of the Application of Computational Fluid Dynamics for Adult Aortic Diseases. Rev. Cardiovasc. Med..

[18] Fantazzini A., Esposito M., Finotello A., Auricchio F., Pane B., Basso C., Spinella G., Conti M. (2020). 3D Automatic Segmentation of Aortic Computed Tomography Angiography Combining Multi-View 2D Convolutional Neural Networks. Cardiovasc. Eng. Technol..

[19] Sturla F., Caimi A., Romarowski R.M., Nano G., Glauber M., Redaelli A., Votta E., Marrocco-Trischitta M.M. (2023). Fast Approximate Quantification of Endovascular Stent Graft Displacement Forces in the Bovine Aortic Arch Variant. J. Endovasc. Ther..

[20] Preventza O., Le Huu A., Olive J., Cekmecelioglu D., Coselli J.S. (2022). Endovascular repair of the ascending aorta: The last frontier. Ann. Cardiothorac. Surg..

